# Characterization of Fluorescent Proteins for Three- and Four-Color Live-Cell Imaging in *S*. *cerevisiae*

**DOI:** 10.1371/journal.pone.0146120

**Published:** 2016-01-04

**Authors:** Ryo Higuchi-Sanabria, Enrique J. Garcia, Delia Tomoiaga, Emilia L. Munteanu, Paul Feinstein, Liza A. Pon

**Affiliations:** 1 Department of Pathology and Cell Biology, Columbia University, New York, NY, United States of America; 2 Herbert Irving Comprehensive Cancer Center, Columbia University, New York, NY, United States of America; 3 Department of Biological Sciences, Hunter College and The Graduate Center Biochemistry, Biology and Biopsychology and Behavioral Neuroscience Programs, CUNY, New York, NY 10065, United States of America; Institute of Biology Valrose, FRANCE

## Abstract

*Saccharomyces cerevisiae* are widely used for imaging fluorescently tagged protein fusions. Fluorescent proteins can easily be inserted into yeast genes at their chromosomal locus, by homologous recombination, for expression of tagged proteins at endogenous levels. This is especially useful for incorporation of multiple fluorescent protein fusions into a single strain, which can be challenging in organisms where genetic manipulation is more complex. However, the availability of optimal fluorescent protein combinations for 3-color imaging is limited. Here, we have characterized a combination of fluorescent proteins, mTFP1/mCitrine/mCherry for multicolor live cell imaging in *S*. *cerevisiae*. This combination can be used with conventional blue dyes, such as DAPI, for potential four-color live cell imaging.

## Introduction

Enhanced Green fluorescent protein (GFP) from *Aequorea victoria* and its other derivatives including cyan and yellow fluorescent proteins (CFP and YFP, respectively) are widely used due to their narrow emission spectra, photostability, and low cellular toxicity [1]. While it is possible to express FP-fusion proteins from plasmids, there is significant cell-to-cell variation in plasmid-borne FP signal strength, largely due to variations in plasmid copy number. In contrast, expression of FP-fusions by tagging well-characterized proteins of interest at their chromosomal locus provides a means to test whether the tag perturbs function, express tagged proteins at endogenous levels and obtain more uniform FP signal within a cell population. We characterized tagging cassettes for insertion of FPs into the yeast genome, demonstrated that the tags can be used for 3- and 4-color imaging in living cells, and describe the benefits of multicolor imaging with these cassettes.

Currently, (BFP)/GFP/RFP [2] or CFP/YFP/RFP [3] are used for three-color live-cell in the yeast system. However, both have limitations. The UV illumination used for imaging of BFP in live cells results in phototoxicity, which in turn leads to organelle fragmentation or rupturing, production of reactive oxygen species, and cell death [4]. In addition, the brightest BFPs available in yeast, mTagBFP1 and mTagBFP2, disrupt function of fusion proteins [2].

Cyan fluorophores, on the other hand, are shifted higher in excitation and emission spectra, making them more amenable to long-term, live-cell imaging. However, cyan is shifted closer to GFP than BFP, which results in bleed-through using most conventional green illumination parameters. While CFP/YFP/RFP can be used for three-color imaging modality, most CFPs and YFPs are derived from GFP. As a result of the high degree of identity in DNA sequences, insertion of all three proteins into the yeast genome by the widely used method of homologous recombination is difficult. Plasmid-borne CFP and YFP fusion proteins can be used for multicolor imaging. However, plasmid-borne tagged proteins exhibit cell-to-cell variation in expression level due to variation in plasmid copy number, which creates challenges for quantitative analysis.

Recent advances have led to the development of FPs that are monomeric and span a broad array of the color spectrum. Moreover, since many of the newly developed FPs are from different cellular sources and are genetically distinct, multiple FPs can be introduced into the same yeast cell by homologous recombination. Here, we characterized tagging cassettes and expanded their utility (i.e. for N-terminal tagging and for usage with alternative selection markers) for three- and four-color live-cell imaging in *S*. *cerevisiae*.

The FPs used for these studies are monomeric teal fluorescent protein 1 (mTFP1), mCitrine, mCherry, and the blue DNA-binding dye, DAPI or the fluorescent protein mTagBFP2. mTFP1 is a derivative of cyan FP484 from the coral *Clavularia sp*. [5]. mTFP1 has many advantages over CFP, including higher photostability, decreased acid sensitivity, and narrower emission spectrum. In addition, mTFP1 is readily visible using standard CFP imaging conditions. A previous study has tested the utility of mTFP1 with other fluorophores for two-color imaging [6]. mCitrine is a variant of YFP [7]. Compared to YFP, mCitrine is less pH or halide sensitive, more photostable and exhibits better expression at 37°C and in organelles. mCherry is a monomeric FP from the coral *Disconsoma sp*. protein mRFP. mCherry matures more rapidly than mRFP. It is also brighter, more amenable to fusions at the N- or C-terminus and more photostable compared to mRFP [8]. While mCherry had sufficient brightness and phostability for our studies, other RFPs have been developed, including mRuby2 and TagRFP-T, which can be brighter and more photostable compared to mCherry [2].

We characterized both C-terminal and N-terminal tagging constructs for mTFP1, and N-terminal constructs for mCitrine and mCherry for yeast multi-color imaging. Since each of these FPs is resolved from blue dyes, they can be used in conjunction with the DNA binding dye DAPI and BFPs, such as mTagBFP2 for three- or four-color imaging.

## Materials and Methods

### Plasmid construction

We created C-terminal and N-terminal mTFP1 tagging vectors by cloning the fluorophore sequences into the previously published pFA6 and pOM series vectors [9, 10]. We also synthesized pOM series vectors with mCitrine and mCherry to make N-terminal tagging with these valuable fluorophores readily available (we did not clone mCitrine and mCherry into pFA6 vectors since C-terminal tagging vectors for these FPs are readily available [11]). These vectors share the same tagging primers as the original pFA6 and pOM vectors and can be used interchangeably with them (S1 Fig).

Yeast tagging plasmids were derived from either pFA6a-GFP(S65T)-kanMX6 or pFA6-GFP(S65T)-HISMX6 [10] for C-terminal tagging constructs and from POM42 and POM43 for N-terminal tagging constructs [9]. To produce the C-terminal tagging constructs, the mTFP1 sequence was obtained from [12] and PCR-amplified with PacI+CAGT on the 5’ end and AscI on the 3’ end. Both the PCR product and parent vectors were digested with PacI and AscI. GFP was then dropped out of the pFA6-GFP vectors containing *HIS-3* or *KanMX-6* selectable markers by gel extraction of the parent vector and replaced with mTFP1. A similar method was employed for the N-terminal constructs, dropping GFP from POM42 or POM43 plasmids using serial digestion with BamHI and SpeI and replacing them with PCR-amplified mTFP1, mCitrine, or mCherry flanked with BamHI and SpeI. Primers used for this study can be found in Table A in S1 File. All plasmids constructed for these studies are accessible at Addgene.

### Yeast strain construction

For construction of strains with fluorescent protein tags, a PCR fragment containing regions homologous to sequences directly upstream and downstream of the stop codon (for C-terminal tags) or MTS cleavage site (for N-terminal tags) and coding regions for the FP and selection marker was amplified from the appropriate plasmids (Table B in S1 File) using the primers listed in Table C in S1 File. BY4741 cells were transformed with the PCR product using a standard lithium acetate transformation method and were selected on either YPD plates with appropriate drug selections or synthetic complete (SC) plates with appropriate amino acid dropouts. Tagging was confirmed by fluorescence microscopy. For N-terminal tags, the selection marker was removed by transforming cells with the pSH62 vector and gal-inducing for 4 hours followed by replica plating on YPD and SC with appropriate dropouts to select for loss of selection marker [9]. The *cit1*∆ strain was synthesized by replacing the *CIT1* gene with *LEU2*. A PCR product with sequences homologous to regions directly upstream of the start codon and downstream of the stop codon and coding regions for *LEU2* was amplified from plasmid POM13 (Addgene, Cambridge, MA) using forward primer 5’ ATAAGGCAAAACATATAGCAATATAATACTATTTACGAAGTGCAGGTCGACAACCCTTAAT 3’ and reverse primer 5’ TTTGAATAGTCGCATACCCTGAATCAAAAATCAAATTTTCCGCAGCGTACGGATATCACCTA 3’. BY4741 cells were transformed with the PCR product using the lithium acetate method and selected on SC-LEU plates. Deletion of *CIT1* was confirmed through PCR using forward primer 5’ CCGATACTATCGACTTATCCGACCTC 3’ and reverse primer 5’ GCCAAGTATATAACATAACCGGTAGGC 3’.

### Microscopy

All wide-field imaging was performed as previously described [13] on an inverted AxioObserver.Z1 microscope with a 100x/1.3 oil EC Plan-Neofluar objective (Zeiss, Thornwood, NY) and Orca ER cooled CCD camera (Hamamatsu, Bridgewater, NJ). For visualization of GFP, mTFP1, mCitrine, mCherry, and DAPI/mTagBFP2, fluorophores were excited by a metal halide lamp using standard GFP (Zeiss filter set 38 HE; excitation 470/40, dichroic FT 495, emission 525/50), CFP (Zeiss filter set 47 HE; excitation 436/25, dichroic FT 455 HE, emission 480/40), YFP (Zeiss filter set 46 HE; excitation 500/25, dichroic FT 515 HE, emission 535/30), rhodamine (Zeiss filter set 43 HE; excitation 550/25, dichroic FT 570, emission 605/70), and DAPI (Zeiss filter set 49; excitation G365, dichroic FT 395, emission 445/50) filter sets, respectively. Hardware was controlled by ZEN (Zeiss) software.

### Staining of mitochondrial DNA (mtDNA) using DAPI

mtDNA was visualized by DAPI staining in live cells (at mid-log OD_600_ = 0.1–0.3) at a final concentration of 1 μg/ml DAPI directly in cell culture media (SC). Cells were stained for 10 min shaking at room temperature, washed three times with cell culture media, and imaged immediately for no longer than 5 min.

### Measurement of fluorescence loss over time

For wide-field microscopy (GFP, mTFP1, mCitrine, and mCherry), cells expressing Cit1p-FP were imaged on an inverted AxioObserver.Z1 microscope using a metal halide lamp and using the appropriate filter wheels as described above. Cells were grown to mid-log (OD_600_ = 0.1–0.3) in liquid SC media, concentrated, mounted on slides with cover slips and imaged for no longer than 10 min. Images were taken across a single plane at the center of the cell at 1 sec intervals for 120 sec using 216 gain and 200 ms exposure time. The integrated fluorescence intensity was measured over time using ImageJ with thresholding. Raw integrated fluorescence intensity at each time point was measured and normalized as the % fluorescence remaining.

### Functional assay of proteins using growth rate

Fluorescent protein tags were tested for functionality by measuring growth rate in liquid culture and solid media. Growth rate in liquid culture was measured by growing cells to mid-log phase in YPD and diluting to an OD_600_ of 0.07. 10 μL of each of the diluted strains was added to each well containing 200 μL of YPD. Cells were propagated in a 96-well plate at 30°C and optical density measurements were made every 20 min for 72 hours using a NanoQuant (Tecan, San Jose, CA). Maximum growth rate (slope) was defined as the greatest change in OD over a 60 min interval in 72 hours.

Growth rate on solid medium was determined by a serial dilution assay. Cells were grown to mid-log phase in YPD and diluted to an OD_600_ of 0.1. 10-fold serial dilutions were prepared in YPD and YPG, and 5 μL was plated on YPD and YPG, respectively. Cells were grown in a 30°C incubator for 3–4 days.

## Results

### mTFP1 can be targeted to mitochondria and does not disrupt mitochondrial function

**Fig 1:**
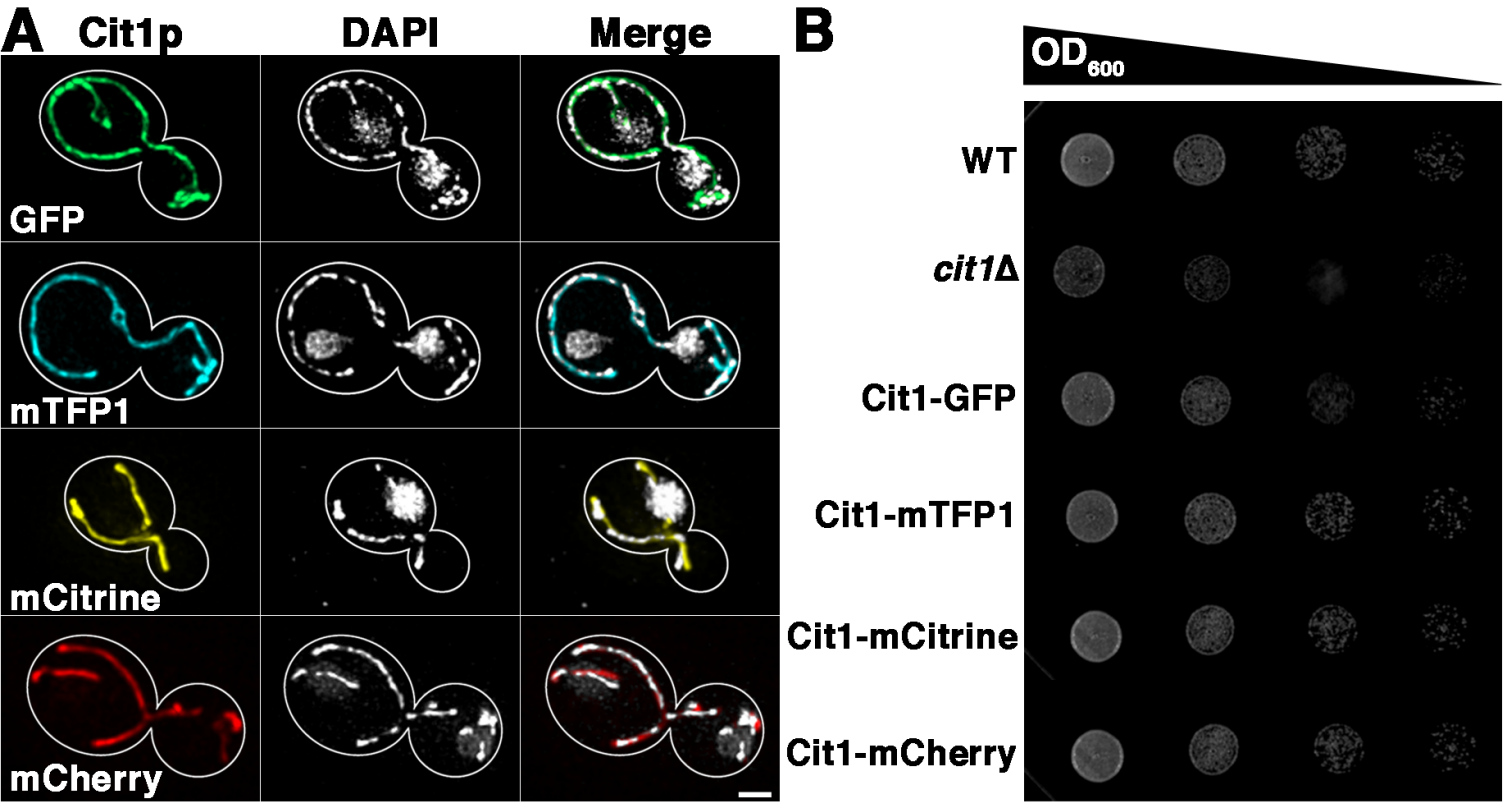
C-terminal tagging of Cit1p with mTFP1 does not disrupt localization or function of the protein. A) BY4741 cells expressing Cit1-GFP, Cit1-mTFP1, Cit1-mCitrine, or Cit1-mCherry were stained with 1 μg/ml DAPI for 10 min as described in Materials and Methods. DAPI-stained cells were imaged on a wide-field microscope. Z-series were collected through the entire cell at 0.5 μm intervals using a metal halide lamp and appropriate filters at 216 gain and 200 ms exposure time. Cell outlines were drawn over phase images. Scale bar = 1 μm. n = nucleus. mtDNA = mitochondrial DNA. B) BY4741 and cit1∆ cells, and BY4741 cells expressing Cit1-GFP, Cit1-mTFP1, Cit1-mCitrine, or Cit1-mCherry were grown to mid-log phase in YPD and diluted to OD_600_ = 0.01 in YPG. 10-fold serial dilutions were performed and 5 μl was placed on solid media (YPG) and grown at 30°C for three days. Images are representative of three independent trials.

Previous studies indicate that tagging of two proteins in the eisosome, a large protein complex that contributes to endocytosis, has no effect on protein function or localization [6]. As a first step to characterize an approach for 3- and 4-color live cell imaging in yeast, we generated C-terminal tagging cassettes for mTFP1 and other FPs and tagged Cit1p (Citrate Synthase 1), an abundant citric acid cycle protein that localizes to the mitochondrial matrix [14], at its C-terminus with mTFP1, GFP, mCitrine, or mCherry. We find that Cit1p that is fused to each of these FPs localizes to structures that resemble mitochondria, i.e. long tubular structures that align along the mother-bud axis and accumulate in the bud tip. To confirm that the labeled structures are mitochondria, we stained the cells with the DNA binding dye DAPI to visualize mitochondrial DNA (mtDNA). We find that all four fusion proteins co-localize with mtDNA (Fig 1A).

We also tested the localization of Cit1p tagged at its N-terminus with mTFP1, mCitrine, and mCherry using our newly synthesized N-terminal tagging constructs. To insure that N-terminally tagged proteins localize to mitochondria, FP tags were inserted in Cit1p between the N-terminal mitochondrial localization sequence and the mature protein. N-terminally tagged mTFP1-Cit1p, mCitrine-Cit1p, and mCherry-Cit1p all localize to mitochondrial structures and co-localize with DAPI (S2A Fig). However, yeast expressing Cit1p that is tagged at its N-terminus with GFP exhibit defects in mitochondrial morphology. Although most of the mitochondria in GFP-mCit1p are long tubular structures, we detect some abnormal, large, spherical mitochondria (S2A Fig).

Next, we tested whether tagging Cit1p affects cellular fitness or protein function. To test for possible effects on cell toxicity, we measured growth rates of yeast cells expressing Cit1p-mTFP1 using glucose as a carbon source. We find that the growth rate of Cit1p-mTFP1 expressing cells using rich, glucose-based media is similar to those of yeast that have no FPs, and of yeast expressing Cit1p-GFP, Cit1p-mCitrine and Cit1p-mCherry (S3 Fig). To determine whether tagging Cit1p with mTFP1 or other FPs affects mitochondria function, we examined the growth rate of these cells grown on rich, glycerol-based media, i.e. under respiration-driven growth conditions (Fig 1B). We confirmed that deletion of *CIT1* results in reduced rates of respiration-driven growth. In addition, we found that the respiration-driven growth rate of yeast expressing any of the C-terminally tagged Cit1p-FPs is similar to that observed in cells that do not express tagged Cit1p. Thus, tagging Cit1p at its C-terminus at its chromosomal locus does not affect yeast cell growth or Cit1p function.

We find similar results with Cit1p tagged at the N-terminus with mTFP1, mCitrine, and mCherry: yeast expressing these Cit1p fusions exhibit normal growth rates when propagated using fermentable or non-fermentable carbon sources. In contrast, yeast expressing Cit1p tagged at the N-terminus with GFP exhibit reduced growth rates on glycerol-based media (S2B–S2D Fig). Thus, C-terminal tagging of Cit1p with the FPs used in this study does not have detectable effects on mitochondrial respiratory activity. However, N-terminal tagging of Cit1p with GFP disrupts the localization and function of Cit1p.

### Properties of mTFP1/mCitrine/mCherry when expressed in living yeast cells

**Fig 2:**
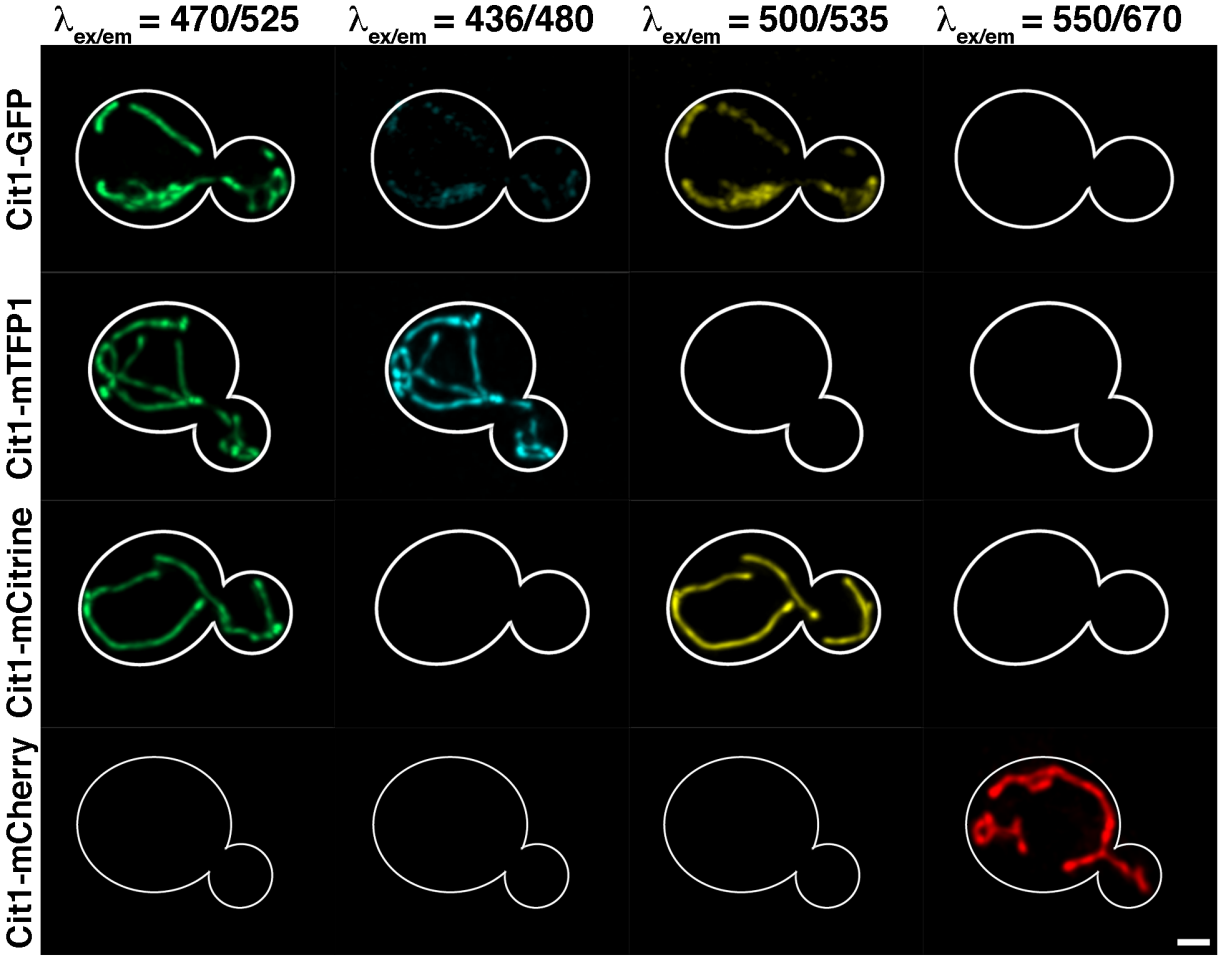
Spectral compatibility of mitochondria-targeted FPs. BY4741 cells expressing Cit1-GFP, Cit1-mTFP1, Cit1-mCitrine, and Cit1-mCherry were imaged on a wide-field microscope. Z-series were collected at 0.5 μm intervals throughout the entire cell using a metal-halide lamp with standard GFP, CFP, YFP, and rhodamine filters using 216 gain and 200 ms exposure times. Full filter specifications are listed in Materials and Methods. Only the center wavelengths for our filters for values of excitation and emission filters are listed for ease of readability.

To determine whether mTFP1, mCitrine and mCherry can be used for simultaneous, 3-color imaging in yeast, we studied whether the fluorescence of these proteins can be resolved using conventional epi-fluorescence filter sets (Fig 2). mTFP1 and mCitrine fusion proteins produce a robust mitochondrial signal under conditions used for GFP imaging (λ_ex/em_ = 470/525). Thus, they cannot be used for multicolor imaging with GFP. However, we do not detect bleed-through between mTFP1, mCitrine, and mCherry under our imaging conditions (λ_ex/em_ = 436/480 for mTFP1; λ_ex/em_ = 500/535 for mCitrine; and λ_ex/em_ = 550/670 for mCherry).

**Fig 3:**
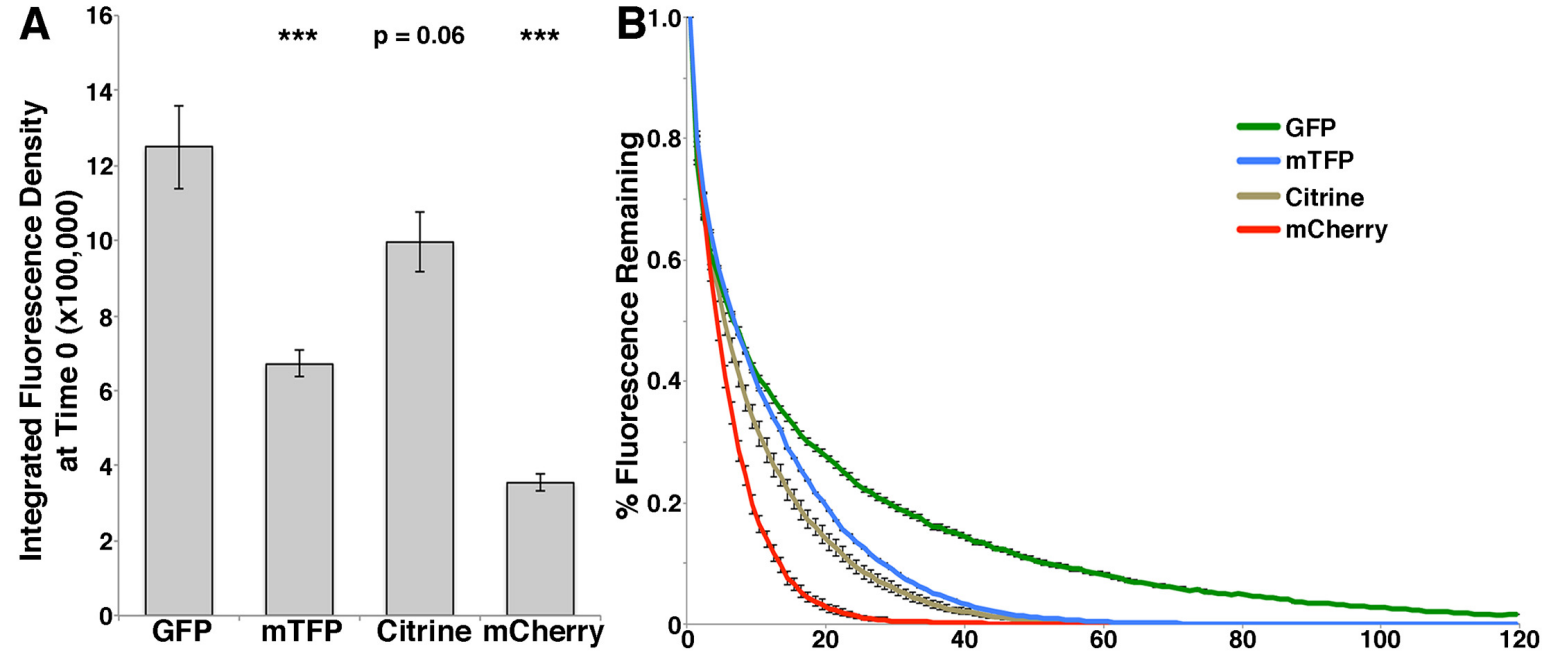
Perceived brightness and stability of mTFP1 relative to GFP, mCitrine, and mCherry. BY4741 cells expressing Cit1-GFP, Cit1-mTFP1, Cit1-mCitrine, or Cit1-mCherry were imaged on a wide-field microscope. Single-plane, time-lapse imaging was performed at the center of the cell at 1 sec intervals for 120 sec using a metal halide lamp and appropriate filters at 216 gain and 200 ms exposure for each fluorophore. A) Integrated fluorescence density at time = 0 was measured using Image J as described in Materials and Methods. *** = p < 0.001. Error bars are SEM. B) Integrated fluorescence density was measured at each time point and normalized to the integrated fluorescence density at time = 0 and graphed as % fluorescence remaining as a function of time. Error bars are SEM. n = 28–44 cells per strain. Data is representative of 3 independent trials.

We next tested the perceived brightness of mTFP1 relative to GFP, mCitrine, and mCherry when expressed in living yeast cells. The intrinsic brightness of an FP, the product of its extinction coefficient and quantum yield, is typically measured using purified proteins. Previous studies revealed that the intrinsic brightness of the FPs of interest for these studies is: mCitrine > mTFP1 > GFP > mCherry (Table 1) [1, 5]. In contrast, the perceived brightness of an FP is determined by factors including the intrinsic brightness, optical properties of the imaging and detection systems, as well as FP expression, folding and context. To determine the perceived brightness of mitochondria-targeted FPs of interest, we performed wide-field, fluorescence imaging of Cit1p tagged with GFP, mTFP1, mCitrine, or mCherry in living yeast cells. Imaging was performed with minimal variability between the acquisition strategies for each fluorophore, utilizing the same metal halide lamp at identical power and filter sets specific to each fluorophore as described in *Materials and Methods*. Although the signal from all 4 fluorophores is sufficient for these studies, we find variability in integrated fluorescence intensity: GFP > mCitrine > mTFP > mCherry (Fig 3A) (Table 1).

**Table 1:** Properties of fluorescent proteins used in this study. Full spectra and information for all fluorescent proteins are available at (http://nic.ucsf.edu/FPvisualization/). λex/em are listed as the peak excitation and emission wavelength of the FP. Intrinsic brightness is derived from the product of its extinction coefficient and quantum yield and higher numbers indicate brighter FPs. Bleaching is listed such that higher numbers indicate more photostable FPs. Perceived brightness is defined by relative integrated intensity versus GFP derived from raw data in Figs 3A and S8B. It is important to note that perceived brightness measurements will depend on the choice of filter sets and the light source (full filter specifications can be found in Materials and Methods). Relative bleaching is defined as the percent fluorescence remaining at time = 20 sec in Fig 3B and in S7C Fig as this was the time point where the greatest difference could be visualized between the fluorophores. mTagBFP2 was not characterized in this study, and its full properties can be found here: [2].

Protein	λ ex (nm)	λ em (nm)	Intrinsic Brightness	Bleaching	Ref.	Perceived Brightness	Relative Bleaching
GFP	488	507	33.6	174	[22]	100	28
mTagBFP2	399	454	32.4	53	[23]	N/A	N/A
mCerulean	433	475	26.7	36	[24]	60	9
mTFP1	462	492	54	N/A	[5]	54	20
mCitrine	516	529	58.5	49	[7]	80	15
mCherry	587	610	15.8	96	[8]	28	3

In addition, we compared fluorescent protein signal compared to background signal for all fluorescent protein fusions used in this study. Using our conventional imaging conditions, there is no detectable autofluorescence of yeast. However, we can induce autofluorescence of organelle or cellular structures using high exposure times or hydrogen peroxide-induced oxidative stress. Under these conditions, we find that GFP-imaging conditions induce autofluorescence of structures reminiscent of mitochondria, mCherry-imaging conditions induce autofluorescence of structures reminiscent of vacuoles, and mTagBFP2-imaging conditions induce autofluorescence of the entire yeast cell (S5 Fig). Therefore, we recommend using mTFP1 or mCitrine for determination of protein localization if high exposure times or stressed conditions are essential.

Finally, we measured the photostability of mitochondria-targeted FPs. Yeast expressing Cit1p that was tagged at its C-terminus with mTFP1, mCitrine or mCherry were imaged along a single plane at the center of the mother cell at 1 sec intervals for 120 sec. We then measured the integrated fluorescence intensity at each time point, normalized it to the integrated fluorescence intensity at time 0, and plotted them as percent fluorescence remaining as a function of time (S4 Fig). We find that mTFP1 is less photostable compared to GFP; however, it is more photostable compared to mCitrine and mCherry under our experimental conditions.

### 3- and 4-color, live-cell imaging using mTFP1/mCitrine/mCherry

**Fig 4:**
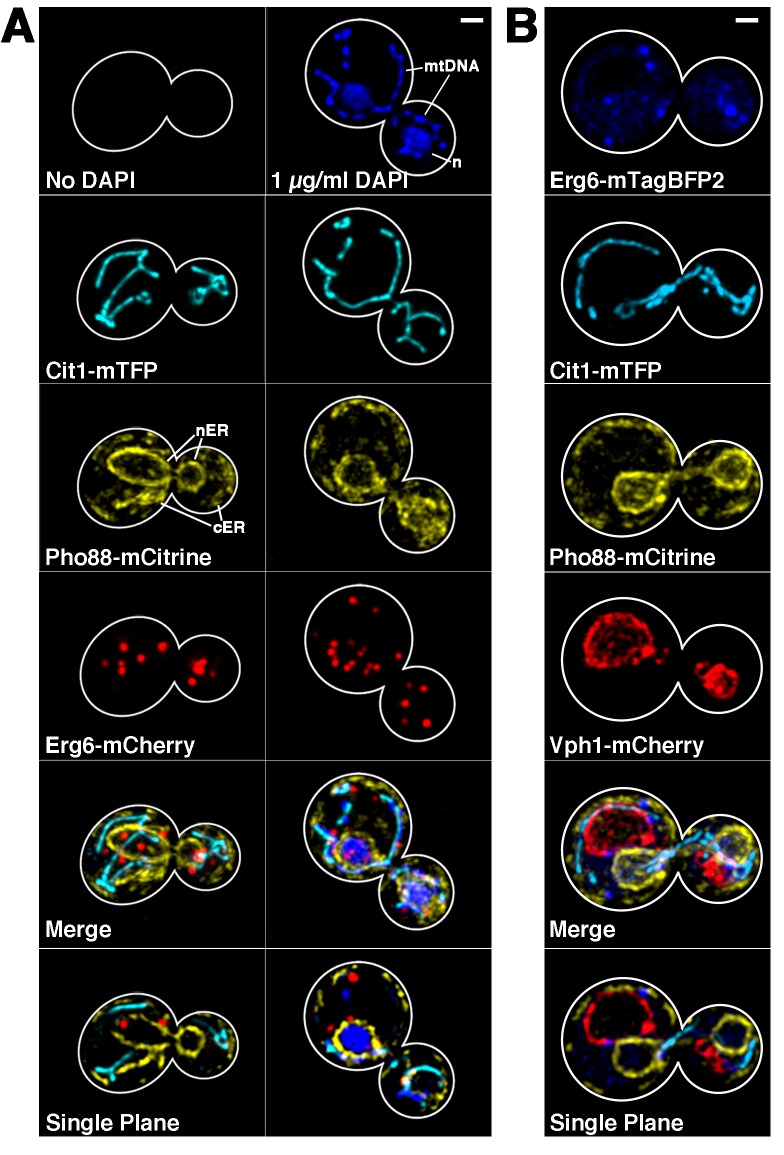
Utilization of mTFP1, mCitrine, mCherry and DAPI for 3- and 4-color, live-cell imaging. A) BY4741 cells expressing Cit1-mTFP1, Pho88-mCitrine, and Erg6-mCh were imaged either untreated (left panels) or after staining with 1 μg/mL DAPI for 10 min (right panel) at 30°C as described in Materials and Methods. B) Images of BY4741 cells expressing Erg6-mTagBFP2, Cit1-mTFP, Pho88-CmCitrine, and Vph1-mCherry. For A-B, Z-series were collected with wide-field microscopy at 0.5 μm intervals throughout the entire cell using a metal-halide lamp and appropriate filters using 216 gain and the following exposure times: 100 ms for Cit1-mTFP1, 200 ms for Pho88-mCitrine, 200 ms for Erg6-mCherry, 100 ms for DAPI, and 400 ms for mTagBFP2. Cell outlines were drawn over phase images. Scale bar = 1 μm. n = nucleus. mtDNA = mitochondrial DNA. nER = nuclear ER. cER = cortical ER.

To determine the utility of mTFP1/mCitrine/mCherry as a viable 3-color imaging modality, we performed multicolor imaging of cells expressing Cit1p-mTFP1, Pho88-mCitrine, and Erg6-mCherry. In all cases, proteins were tagged by insertion of the FP into the chromosomal locus of the gene of interest at its C-terminus. As described above, Cit1p is a tricarboxylic acid cycle protein targeted to the mitochondrial matrix. Pho88p is an ER membrane protein involved in phosphate transport [15]. Erg6p is a methyltransferase that localizes to lipid droplets in yeast [16]. Previous studies revealed that fusion of FPs to the C-termini of these organelle marker proteins does not affect protein function or localization [16–18]. Indeed, expression of Pho88p-mCitrine results in labeling of ER associated with the nuclear envelope (nuclear ER) and ER sheets and tubules underlying the plasma membrane (cortical ER). Tagging of Erg6p with mCherry produces punctate structures, reminiscent of lipid droplet structures. We find that simultaneous expression of mTFP1, mCitrine, and mCherry in a single cell has no significant effect on growth rate in glucose or glycerol-based carbon sources (S6 Fig). More importantly, we find that the FP fusion proteins are targeted to their distinct organelle structures, does not have any obvious effect on organelle morphology and can be resolved spectrally through wide-field microscopy (Fig 4A, left panels).

We also find that mTFP1 can be spectrally resolved from the blue fluorescent DNA binding dye DAPI (Fig 4A, right panels). Cells expressing Cit1p-mTFP1 show no fluorescence with our DAPI imaging conditions (λ_ex/em_ = 365/445), while those stained with DAPI show robust staining of nuclear and mitochondrial DNA. Thus mTFP1/mCitrine/mCherry can be used in combination with blue fluorophores for reliable 4-color, live-cell imaging. However, blue fluorophores require UV illumination, which can cause phototoxicity, and should not be used for long-term imaging.

Previous 3-color imaging studies utilized mTagBFP2. Therefore, we studied fusion proteins tagged with mTagBFP2 alone and in conjunction with the multicolor labeling with mTFP1, mCitrine and mCherry. Yeast expressing Cit1p tagged at its C-terminus with mTagBFP2 exhibit normal growth rates using glucose as a carbon source. However, they exhibit defects in respiration driven growth. (S7 Fig). Thus, we confirmed previous findings that mTagBFP2 tags can compromise protein function [2]. Moreover, we carried out 4-color imaging of yeast expressing Erg6p-mTagBFP2, Cit1p-mTFP1, Pho88p-mCitrine and Vph1p-mCherry. We confirmed that tagging of Vph1p, a subunit of the vacuolar-ATPase complex, results in labeling of structures that resemble vacuoles [19, 20]. We also find that Erg6p-mTagBFP2 localizes to structures that resemble lipid droplets, has no obvious effect on lipid droplet morphology or abundance and is spectrally resolved from mTFP1, mCitrine and mCherry. However, mTagBFP2 has low signal and requires high exposure times using conventional DAPI imaging conditions, which results in autofluorescence of yeast cells (S5 Fig). Thus, mTagBFP2 can be used with mTFP1, mCitrine and m Cherry for 4-color imaging. However, it has limitations for multicolor imaging including possible effects on fusion protein function, weak signal, high background and damage due to illumination conditions.

An alternative strategy for 3-color imaging utilizes CFPs, such as mCerulean, in conjunction with YFPs and RFPs. However, since CFPs and YFPs are both derived from GFP, they have near-identical sequences, making simultaneous expression of both into a single yeast strain using homologous recombination challenging. More importantly, we find that CFP cannot be used in conjunction with conventional blue dyes (S8A Fig). Cells expressing Cit1p-mCerulean, show some fluorescent signal under our DAPI imaging conditions. Moreover, although mCerulean and mTFP1 are similar in perceived brightness, it has lower photostability compared to mTFP1 (S8B and S8C Fig) (Table 1). These data provide additional evidence that mTFP1 provides a viable alternative to CFP for utility in four-color imaging with blue dyes and FPs, and when simultaneous expression of CFPs and YFPs in a single strain is a challenge.

## Discussion

We assessed whether mTFP1, mCitrine and mCherry could be used for three-color, live cell imaging in yeast. We find that tagging an abundant mitochondrial protein, Cit1p with each of these fluorescent proteins at its C- or N-terminus has no effect on cell growth rates or on the localization or function of the protein. Although tagging of Cit1p at its C-terminus with GFP has no obvious detrimental effect, tagging Cit1p at its N-terminus does affect respiration-driven yeast cell growth, presumably though effects on Cit1p function. This is particularly surprising since use of mCitrine, a GFP derivative, to tag Cit1p at its N-terminus has no obvious effect on respiration-driven growth.

To determine whether these FPs are useful for 3-color imaging, we tested whether they can be resolved spectrally and measured the brightness and photostability of Cit1p-FPs within the context of living yeast cells. The peak excitation and emission wavelengths for these FPs are shown (Table 1). We identified conditions using conventional filter sets that resolve each of these FPs. We also find that all of these fluorophores have significant signal intensity above autofluorescence of cellular structures and yeast cells.

The perceived brightness and photostability of mTFP1, mCitrine, or mCherry is less than that of GFP, but sufficient for multi-color fluorescence microscopy. Interestingly, the perceived brightness of Cit1p tagged at its C-terminus measured in living cells (GFP>mCitrine>mTFP1>mCherry) is different from the intrinsic brightness of purified FPs (mCitrine>mTFP1>GFP>mCherry) (Table 1). The photostability of Cit1p-FP in living cells (GFP>mTFP1>mCitrine>mCherry) is also distinct from the photostablity of purified FPs (GFP>mCherry>mCitrine) (Table 1). Since mTFP1 is brighter and bleaches at a much lower rate than mCherry, mTFP1 may be useful in two-color imaging applications in conjunction with mCitrine, when the signal from mCherry-tagged structures is limited.

We also carried 3- and 4-color imaging using mTFP1, mCitrine, and mCherry in living yeast cells. We tagged marker proteins for mitochondria, ER and lipid droplets at their chromosomal loci with these FPs. We found that simultaneous expression of 3 FP-tagged proteins has no significant effect on yeast growth rates or the morphology of organelles visualized, and that mTFP1, mCitrine, and mCherry can be used for robust 3-color live-cell imaging alone and in conjunction with blue dyes and fluorophores, such as DAPI and mTagBFP2.

While BFPs can be used for multicolor imaging, they can have limitations including the requirement of UV illumination, limited availability, negative effects on protein function, and overlap with commonly used blue dyes, such as DAPI. Indeed, we find that tagging with mTagBFP2 perturbs Cit1p function, consistent with previous reports that tagging other proteins with this FP compromises function [2]. Thus, simultaneous imaging with the tagging cassettes for mTFP1, mCitrine and mCherry has clear advantages over multi-color imaging with BFP (e.g. BFP/GFP/mCherry).

Moreover, most CFPs and YFPs have near-identical sequences, making simultaneous expression of both in a single strain difficult. In contrast, since there is no significant sequence homology between of mTFP1, mCitrine and mCherry, these FPs can readily be inserted into the genome of a single yeast cell by homologous recombination and the fluorescent protein can be readily distinguished using antibodies. In addition, mTFP1 has higher photostability and a narrower emission spectrum compared to CFPs [21]. Here we confirmed in live cells that mTFP1 is more photostable than mCerulean, and has a similar perceived brightness. Finally, unlike CFPs, mTFP1 does not overlap with conventional BFP imaging conditions, making them viable for four-color, live cell imaging with blue dyes and FPs. Thus, mTFP1/mCitrine/mCherry 3-color imaging is also an improvement over imaging with multiple GFP-derived proteins, and mTagBFP2/mTFP1/mCitrine/mCherry provides a plausible four-color imaging modality for proteins whose functions are not essential or whose functions are not disrupted by mTagBFP2-tagging.

Overall, we find that mTFP1/mCitrine/mCherry is a proficient 3-color live-cell imaging modality, and can be used with blue dyes and fluorophores for 4-color live-cell imaging. The availability of a robust 3- and 4-color, live-cell imaging modality will expand yeast microscopy and enable complex co-localization experiments.

## Supporting Information

S1 Fig Key components of pFA6 and pOM series vectors. A) Plasmid map of the pFA6-series vectors. pFA6-mTFP1 constructs were synthesized as described in *Materials and Methods* by replacing GFP with mTFP1. The start codon was removed and replaced with CAGT to keep the fluorophore sequence in frame. F1: 5’[gene-specific sequence]CGGATCCCCGGGTTAATTAA3’ R1: 5’[gene-specific sequence]GAATTCGAGCTCGTTTAAAC3’. B) Plasmid map of pOM-series vectors. pOM-mTFP1, pOM-mCitrine, and pOM-mCherry constructs were synthesized as described in *Materials and Methods* by replacing GFP with mTFP1, mCitrine, or mCherry. F2: 5’[gene-specific sequence] GCTGCAGGTCGACAACCCTTAAT3’ R2: 5’[gene-specific sequence] GCGGCCGCATAGGCGACT3’. These tagging vectors are available on Addgene.
(TIF)

S2 Fig Characterization of localization and function of cells expressing GFP-Cit1p, mTFP1-Cit1p, mCitrine-Cit1p, and mCherry-Cit1p. A) BY4741 cells expressing GFP-Cit1, mTFP1-Cit1, mCitrine-Cit1, or mCherry-Cit1 were stained with 1 μg/ml DAPI for 10 min as described in *Materials and Methods*. DAPI-stained cells were imaged on a wide-field microscope. Z-series were collected through the entire cell at 0.5 μm intervals using a metal halide lamp and appropriate filters at 216 gain and 200 ms exposure time. Cell outlines were drawn over phase images. Scale bar = 1 μm. B) BY4741 and *cit1*∆ cells, and BY4741 cells expressing GFP, mTFP1-Cit1, mCitrine-Cit1, or mCherry-Cit1 were grown to mid-log phase in YPD and diluted to OD_600_ = 0.01. 10-fold serial dilutions were performed and 5 μl was placed on solid media (YPD) and grown at 30°C for 3 days. Images are representative of 3 independent trials. C) Growth rates of BY4741 and *cit1*∆ cells, and BY4741 cells expressing GFP, mTFP1-Cit1, mCitrine-Cit1, or mCherry-Cit1 were measured in liquid YPD media as described in *Materials and Methods*. OD_600_ measurements were taken every 20 min and plotted as a function of time. D) Maximum growth rate was defined as the max slope (or greatest change in OD_600_) within a 1 hr period. Error bars represent SEM. n = 10 wells per strain. Data is representative of 3 independent trials.
(TIF)

S3 Fig Growth rates of cells expressing FP-tagged Cit1p. Growth rates of BY4741 and *cit1*∆ cells, and BY4741 cells expressing Cit1-GFP, Cit1-mTFP1, Cit1-mCitrine, and Cit1-mCherry were measured in liquid YPD media as described in *Materials and Methods*. A) OD_600_ measurements were taken every 20 min and plotted as a function of time. B) Maximum growth rate was defined as the max slope (or greatest change in OD_600_) within a one-hour period. Error bars represent SEM. n = 10 wells per strain. Data is representative of 3 independent trials. C) BY4741 and *cit1*∆ cells, and BY4741 cells expressing Cit1-GFP, Cit1-mTFP1, Cit1-mCitrine, or Cit1-mCherry were grown to mid-log phase in YPD and diluted to OD_600_ = 0.01. 10-fold serial dilutions were performed and 5 μl was placed on solid media (YPD) and grown at 30°C for 3 days. Images are representative of 3 independent trials.
(TIF)

S4 Fig Raw data of stability of mTFP1 relative to GFP, mCitrine, and mCherry. BY4741 cells expressing Cit1-GFP, Cit1-mTFP1, Cit1-mCitrine, or Cit1-mCherry were imaged on a wide-field microscope as per Fig 3. Raw data of integrated fluorescence density was plotted over time.
(TIF)

S5 Fig Perceived brightness of fluorescent proteins relative to background and autofluorescence. BY4741 cells expressing Cit1-GFP, Cit1-mTFP1, Cit1-mCitrine, or Cit1-mCherry were imaged on a wide-field microscope. Z-series were collected with wide-field microscopy at 0.5 μm intervals throughout the entire cell using a metal-halide lamp and appropriate filters using 216 gain and similar exposure times for all channels: we used 200 ms exposure time for Cit1-GFP, Cit1mTFP1, Cit1-mCitrine, and Cit1-mCherry. We used 400 ms for Cit1-mTagBFP2 due to lack of signal at 200 ms exposure times. For imaging of background, BY4741 cells expressing no tags were imaged at 200 ms exposure times for all channels for low exposure, 1000 ms exposure times for high exposure and for hydrogen peroxide (H_2_O_2_)-treated cells. For H_2_O_2_ treatment, BY4741 cells were treated with 5 mM H_2_O_2_ for 30 min prior to imaging. Images presented here are raw images with no deconvolution and minimal contrasting to equal levels (white point set at 100 and black point set at 1500 using Volocity image enhancement software) for ease of comparison. Cit1p-Tag cells (left panel) are max projections, and background cells are single-slices at the center of the cell to minimize out-of-focus light for ease of background and autofluorescence visualization. Cell outlines were drawn over phase images. Scale bar = 1 μm.
(TIF)

S6 Fig Growth rates of cells expressing Cit1-mTFP1, Pho88-mCitrine, and Erg6-mCherry. Growth rates of BY4741 and cells expressing Cit1-mTFP1, Pho88-mCitrine, and Erg6-mCherry (Triple-Tag) were measured as described in *Materials and Methods*. A) BY4741 and *cit1*∆ cells, and BY4741 cells expressing Cit1-GFP, Cit1-mTFP1, Cit1-mCitrine, or Cit1-mCherry were grown to mid-log phase in YPD and diluted to OD_600_ = 0.01. 10-fold serial dilutions were performed and 5 μl was placed on solid media (YPD) and grown at 30°C for three days. Images are representative of three independent trials. B) OD_600_ measurements were taken every 20 min and plotted as a function of time. C) Maximum growth rate was defined as the max slope (or greatest change in OD_600_) within a one-hour period. Error bars represent SEM. n = 10 wells per strain. Data is representative of 3 independent trials.
(TIF)

S7 Fig Growth rates of cells expressing Cit1-mTagBFP2. Growth rates of BY4741 and cells expressing Cit1-mTagBFP2 were measured as described in *Materials and Methods*. A) BY4741 and *cit1*∆ cells, and BY4741 cells expressing Cit1-mTagBFP2 were grown to mid-log phase in YPD and diluted to OD_600_ = 0.01. 10-fold serial dilutions were performed and 5 μl was placed on solid media (YPD) and grown at 30°C for three days. Images are representative of three independent trials. B) OD_600_ measurements were taken every 20 min and plotted as a function of time. C) Maximum growth rate was defined as the max slope (or greatest change in OD_600_) within a one-hour period. Error bars represent SEM. n = 10 wells per strain. Data is representative of 3 independent trials.
(TIF)

S8 Fig Comparative analysis of mCerulean and mTFP1 in live yeast cells. (A) BY4741 cells expressing Cit1-mCerulean or Cit1-mTFP1 were imaged on a wide-field microscope. Z-series were collected at 0.5 μm intervals throughout the entire cell using a metal-halide lamp with standard CFP and DAPI filters using 216 gain and 200 ms exposure times. Full filter specifications are listed in *Materials and Methods*. Only the center wavelengths of our filter settings for values of excitation and emission filters are listed for ease of readability. Scale bar is 1 μm. (B-C) BY4741 cells expressing Cit1-mTFP1 or Cit1-mCerulean were imaged on a wide-field microscope. Single-plane, time-lapse imaging was performed at the center of the cell at 1 sec intervals for 120 sec using a metal halide lamp and CFP filters at 216 gain and 200 ms exposure for each fluorophore. B) Integrated fluorescence density at time = 0 was measured using Image J as described in Materials and Methods. *** = p < 0.001. Error bars are SEM. C) Integrated fluorescence density was measured at each time point and normalized to the integrated fluorescence density at time = 0 and graphed as % fluorescence remaining as a function of time. Error bars are SEM. n = 35–39 cells per strain. Data is representative of 3 independent trials.
(TIF)

S1 File Supplemental Tables. Table A. Primers used to synthesize tagging vectors. Table B. Tagging vectors used in this study. Vectors synthesized in this study are available on Addgene. Table C. Primers used in this study to synthesize fluorescent protein fusions. Table D. Strains used in this study.
(DOCX)

## Abbreviations

FP: fluorescent protein
GFP: green fluorescent protein
BFP: blue fluorescent protein
mTFP1: monomeric teal fluorescent protein 1
CFP: cyan fluorescent protein
YFP: yellow fluorescent protein
RFP: red fluorescent protein
mtDNA: mitochondrial DNA
cER: cortical endoplasmic reticulum
nER: nuclear endoplasmic reticulum
