# Supplementary figures and images for: Characterization of Fluorescent Proteins for Three- and Four-Color Live-Cell Imaging in S. cerevisiae
Source: PLoS One. 2016 Jan 4;11(1):e0146120. doi: 10.1371/journal.pone.0146120 (PMC4699809; doi:10.1371/journal.pone.0146120)

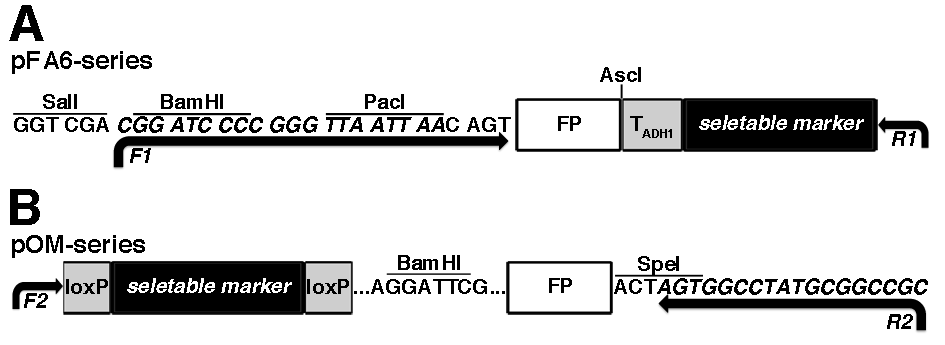

Supplement: S1 Fig — A) Plasmid map of the pFA6-series vectors. pFA6-mTFP1 constructs were synthesized as described in Materials and Methods by replacing GFP with mTFP1. The start codon was removed and replaced with CAGT to keep the fluorophore sequence in frame. F1: 5’[gene-specific sequence]CGGATCCCCGGGTTAATTAA3’ R1: 5’[gene-specific sequence]GAATTCGAGCTCGTTTAAAC3’. B) Plasmid map of pOM-series vectors. pOM-mTFP1, pOM-mCitrine, and pOM-mCherry constructs were synthesized as described in Materials and Methods by replacing GFP with mTFP1, mCitrine, or mCherry. F2: 5’[gene-specific sequence] GCTGCAGGTCGACAACCCTTAAT3’ R2: 5’[gene-specific sequence] GCGGCCGCATAGGCGACT3’. These tagging vectors are available on Addgene. (TIF) [file pone.0146120.s001.tif]

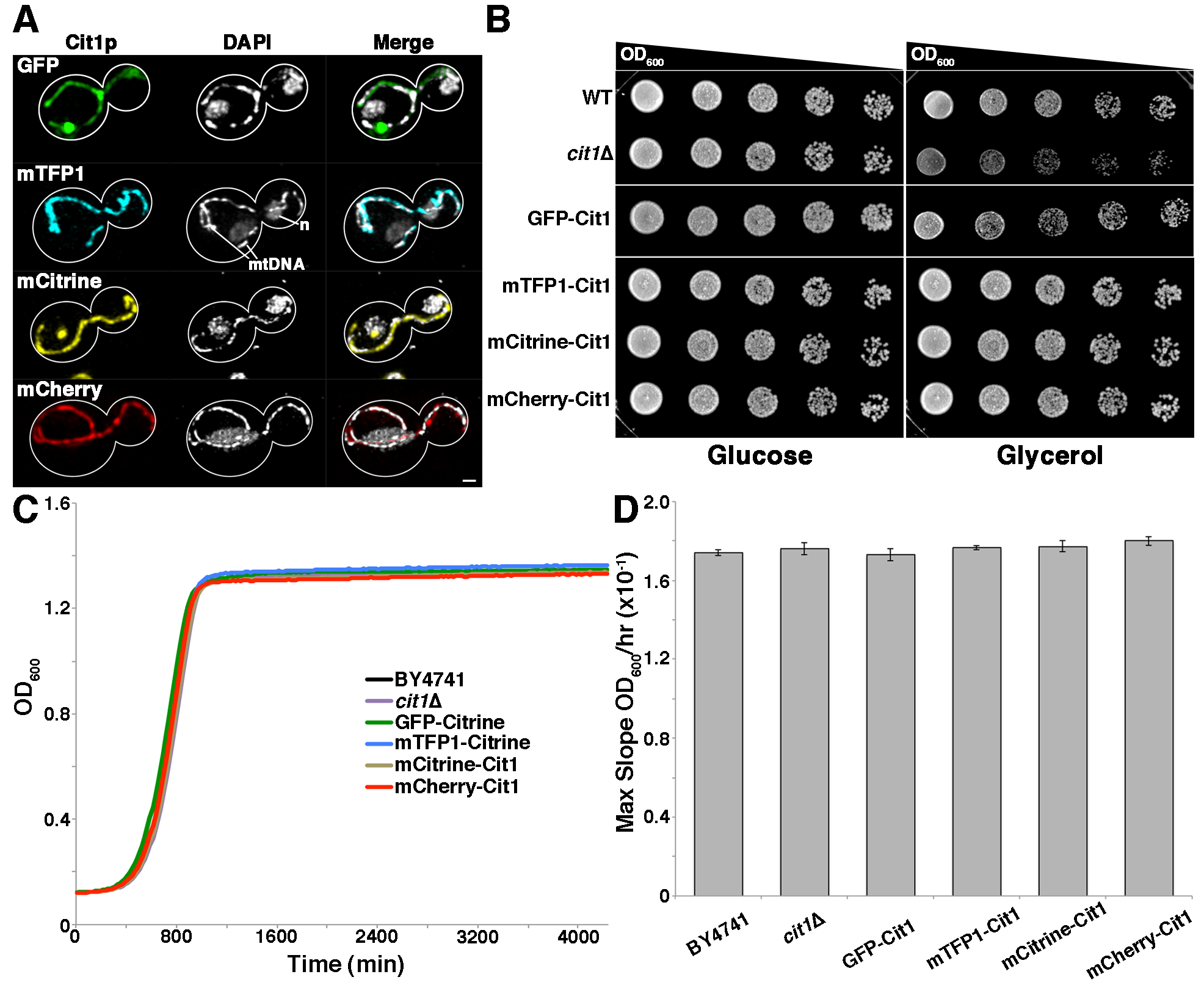

Supplement: S2 Fig — A) BY4741 cells expressing GFP-Cit1, mTFP1-Cit1, mCitrine-Cit1, or mCherry-Cit1 were stained with 1 μg/ml DAPI for 10 min as described in Materials and Methods. DAPI-stained cells were imaged on a wide-field microscope. Z-series were collected through the entire cell at 0.5 μm intervals using a metal halide lamp and appropriate filters at 216 gain and 200 ms exposure time. Cell outlines were drawn over phase images. Scale bar = 1 μm. B) BY4741 and cit1∆ cells, and BY4741 cells expressing GFP, mTFP1-Cit1, mCitrine-Cit1, or mCherry-Cit1 were grown to mid-log phase in YPD and diluted to OD600 = 0.01. 10-fold serial dilutions were performed and 5 μl was placed on solid media (YPD) and grown at 30°C for 3 days. Images are representative of 3 independent trials. C) Growth rates of BY4741 and cit1∆ cells, and BY4741 cells expressing GFP, mTFP1-Cit1, mCitrine-Cit1, or mCherry-Cit1 were measured in liquid YPD media as described in Materials and Methods. OD600 measurements were taken every 20 min and plotted as a function of time. D) Maximum growth rate was defined as the max slope (or greatest change in OD600) within a 1 hr period. Error bars represent SEM. n = 10 wells per strain. Data is representative of 3 independent trials. (TIF) [file pone.0146120.s002.tif]

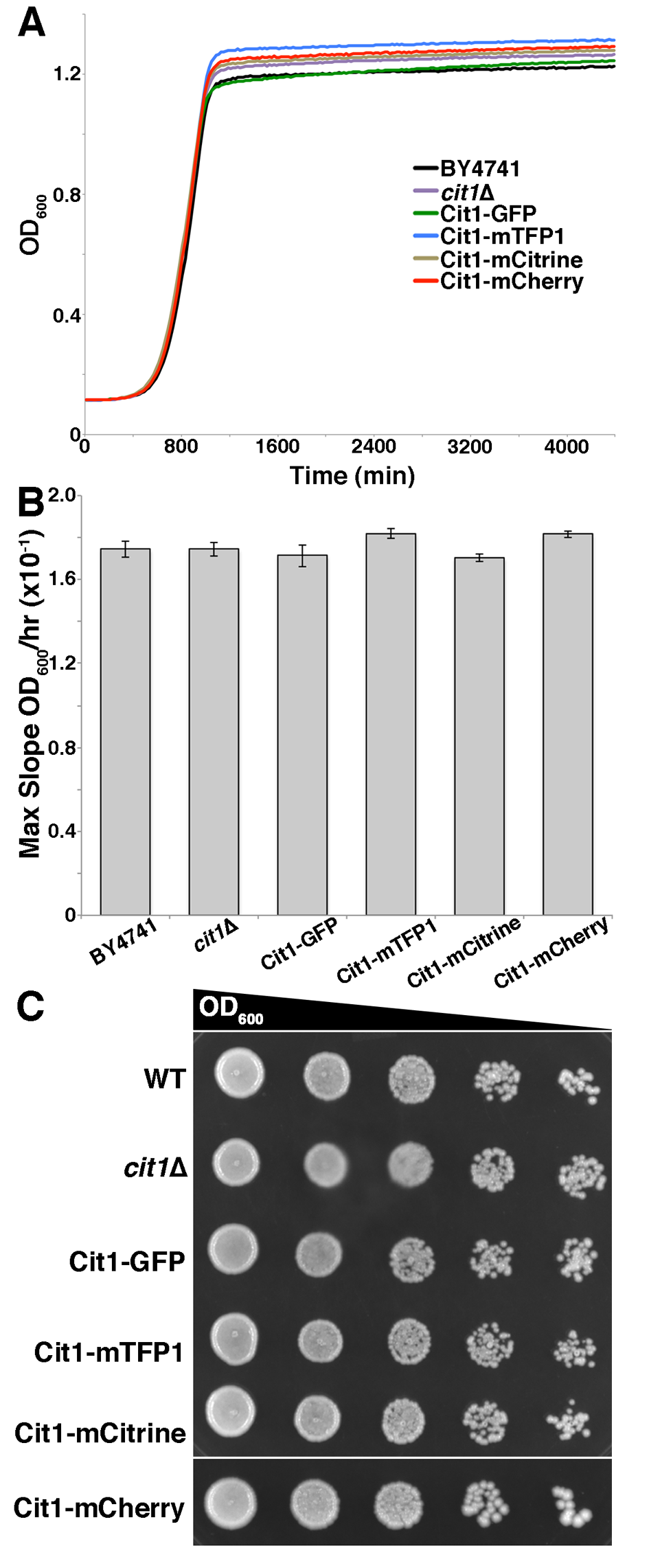

Supplement: S3 Fig — Growth rates of BY4741 and cit1∆ cells, and BY4741 cells expressing Cit1-GFP, Cit1-mTFP1, Cit1-mCitrine, and Cit1-mCherry were measured in liquid YPD media as described in Materials and Methods. A) OD600 measurements were taken every 20 min and plotted as a function of time. B) Maximum growth rate was defined as the max slope (or greatest change in OD600) within a one-hour period. Error bars represent SEM. n = 10 wells per strain. Data is representative of 3 independent trials. C) BY4741 and cit1∆ cells, and BY4741 cells expressing Cit1-GFP, Cit1-mTFP1, Cit1-mCitrine, or Cit1-mCherry were grown to mid-log phase in YPD and diluted to OD600 = 0.01. 10-fold serial dilutions were performed and 5 μl was placed on solid media (YPD) and grown at 30°C for 3 days. Images are representative of 3 independent trials. (TIF) [file pone.0146120.s003.tif]

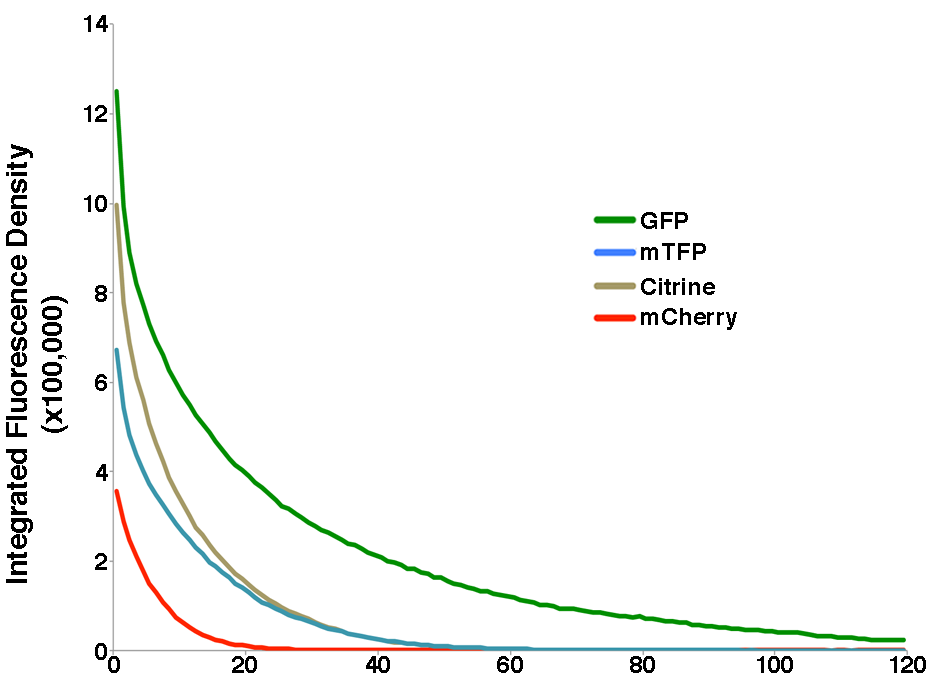

Supplement: S4 Fig — BY4741 cells expressing Cit1-GFP, Cit1-mTFP1, Cit1-mCitrine, or Cit1-mCherry were imaged on a wide-field microscope as per Fig 3. Raw data of integrated fluorescence density was plotted over time. (TIF) [file pone.0146120.s004.tif]

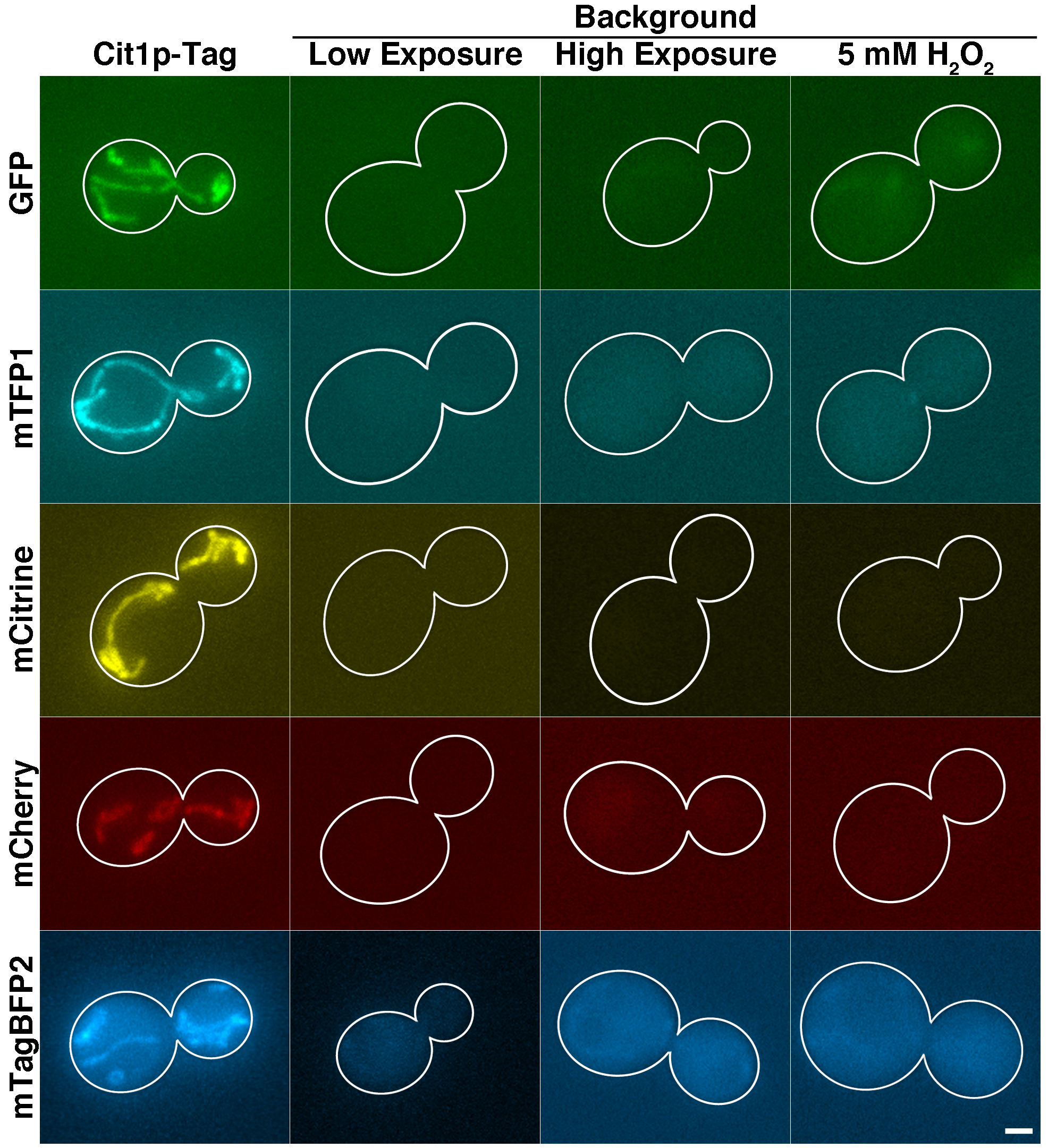

Supplement: S5 Fig — BY4741 cells expressing Cit1-GFP, Cit1-mTFP1, Cit1-mCitrine, or Cit1-mCherry were imaged on a wide-field microscope. Z-series were collected with wide-field microscopy at 0.5 μm intervals throughout the entire cell using a metal-halide lamp and appropriate filters using 216 gain and similar exposure times for all channels: we used 200 ms exposure time for Cit1-GFP, Cit1mTFP1, Cit1-mCitrine, and Cit1-mCherry. We used 400 ms for Cit1-mTagBFP2 due to lack of signal at 200 ms exposure times. For imaging of background, BY4741 cells expressing no tags were imaged at 200 ms exposure times for all channels for low exposure, 1000 ms exposure times for high exposure and for hydrogen peroxide (H2O2)-treated cells. For H2O2 treatment, BY4741 cells were treated with 5 mM H2O2 for 30 min prior to imaging. Images presented here are raw images with no deconvolution and minimal contrasting to equal levels (white point set at 100 and black point set at 1500 using Volocity image enhancement software) for ease of comparison. Cit1p-Tag cells (left panel) are max projections, and background cells are single-slices at the center of the cell to minimize out-of-focus light for ease of background and autofluorescence visualization. Cell outlines were drawn over phase images. Scale bar = 1 μm. (TIF) [file pone.0146120.s005.tif]

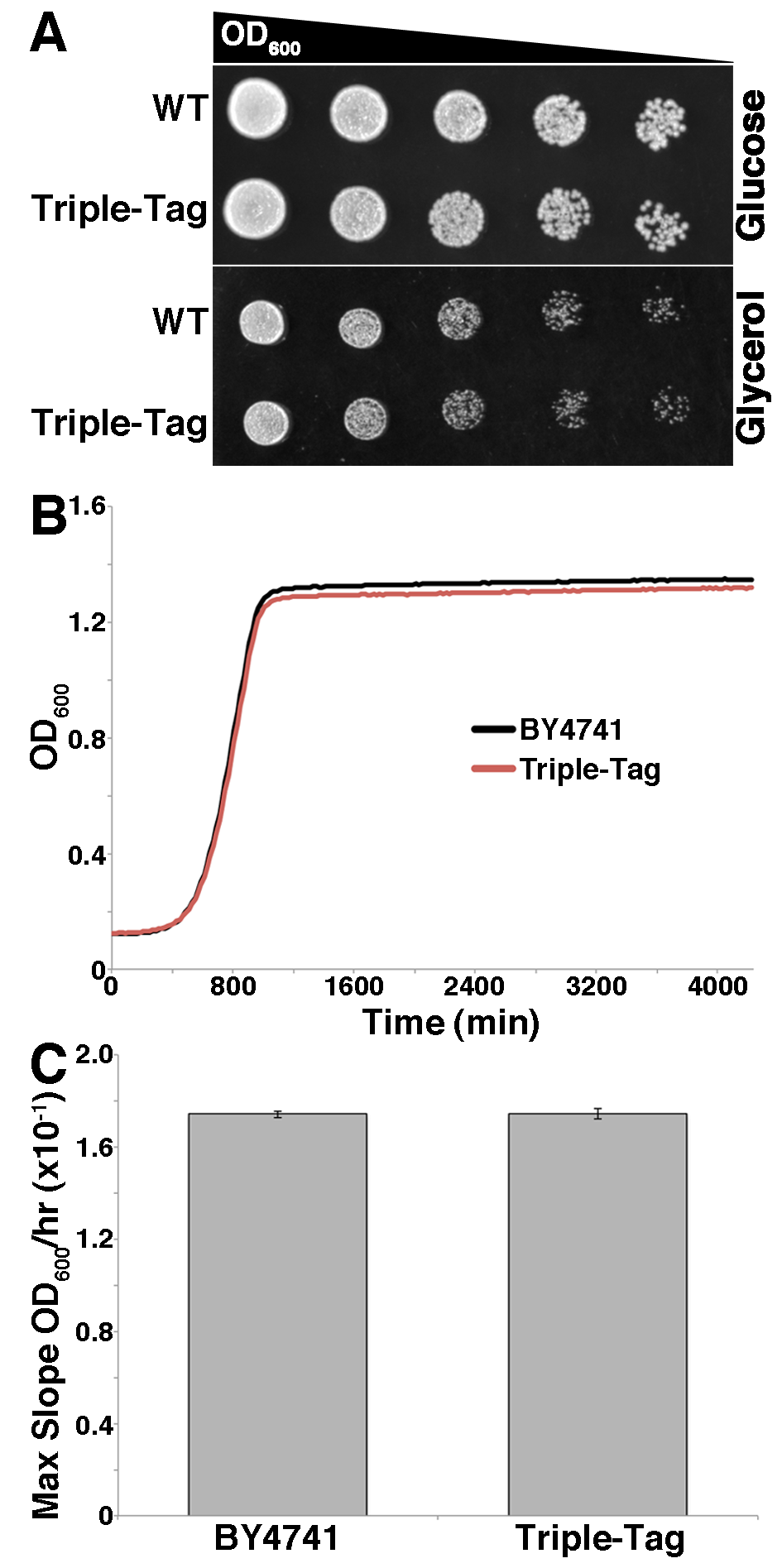

Supplement: S6 Fig — Growth rates of BY4741 and cells expressing Cit1-mTFP1, Pho88-mCitrine, and Erg6-mCherry (Triple-Tag) were measured as described in Materials and Methods. A) BY4741 and cit1∆ cells, and BY4741 cells expressing Cit1-GFP, Cit1-mTFP1, Cit1-mCitrine, or Cit1-mCherry were grown to mid-log phase in YPD and diluted to OD600 = 0.01. 10-fold serial dilutions were performed and 5 μl was placed on solid media (YPD) and grown at 30°C for three days. Images are representative of three independent trials. B) OD600 measurements were taken every 20 min and plotted as a function of time. C) Maximum growth rate was defined as the max slope (or greatest change in OD600) within a one-hour period. Error bars represent SEM. n = 10 wells per strain. Data is representative of 3 independent trials. (TIF) [file pone.0146120.s006.tif]

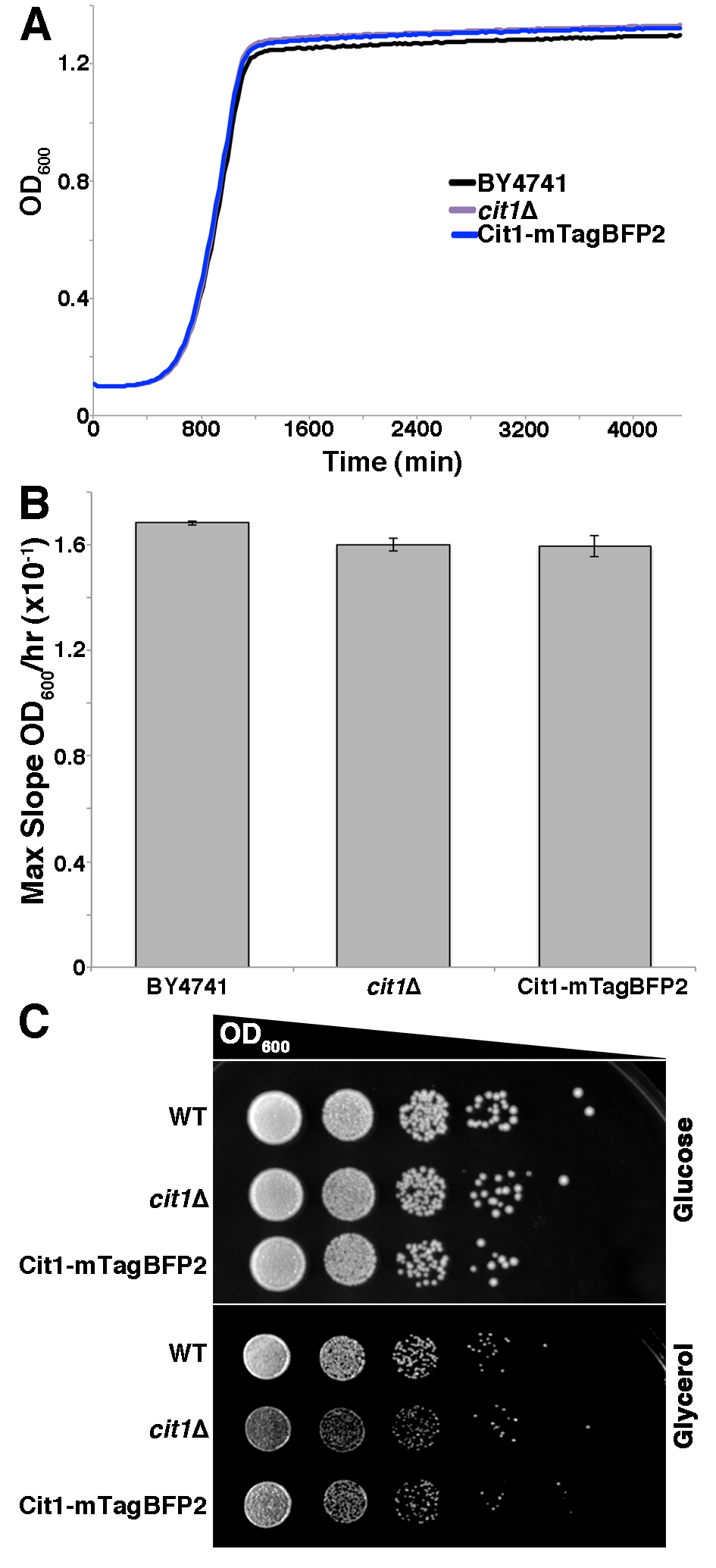

Supplement: S7 Fig — Growth rates of BY4741 and cells expressing Cit1-mTagBFP2 were measured as described in Materials and Methods. A) BY4741 and cit1∆ cells, and BY4741 cells expressing Cit1-mTagBFP2 were grown to mid-log phase in YPD and diluted to OD600 = 0.01. 10-fold serial dilutions were performed and 5 μl was placed on solid media (YPD) and grown at 30°C for three days. Images are representative of three independent trials. B) OD600 measurements were taken every 20 min and plotted as a function of time. C) Maximum growth rate was defined as the max slope (or greatest change in OD600) within a one-hour period. Error bars represent SEM. n = 10 wells per strain. Data is representative of 3 independent trials. (TIF) [file pone.0146120.s007.tif]

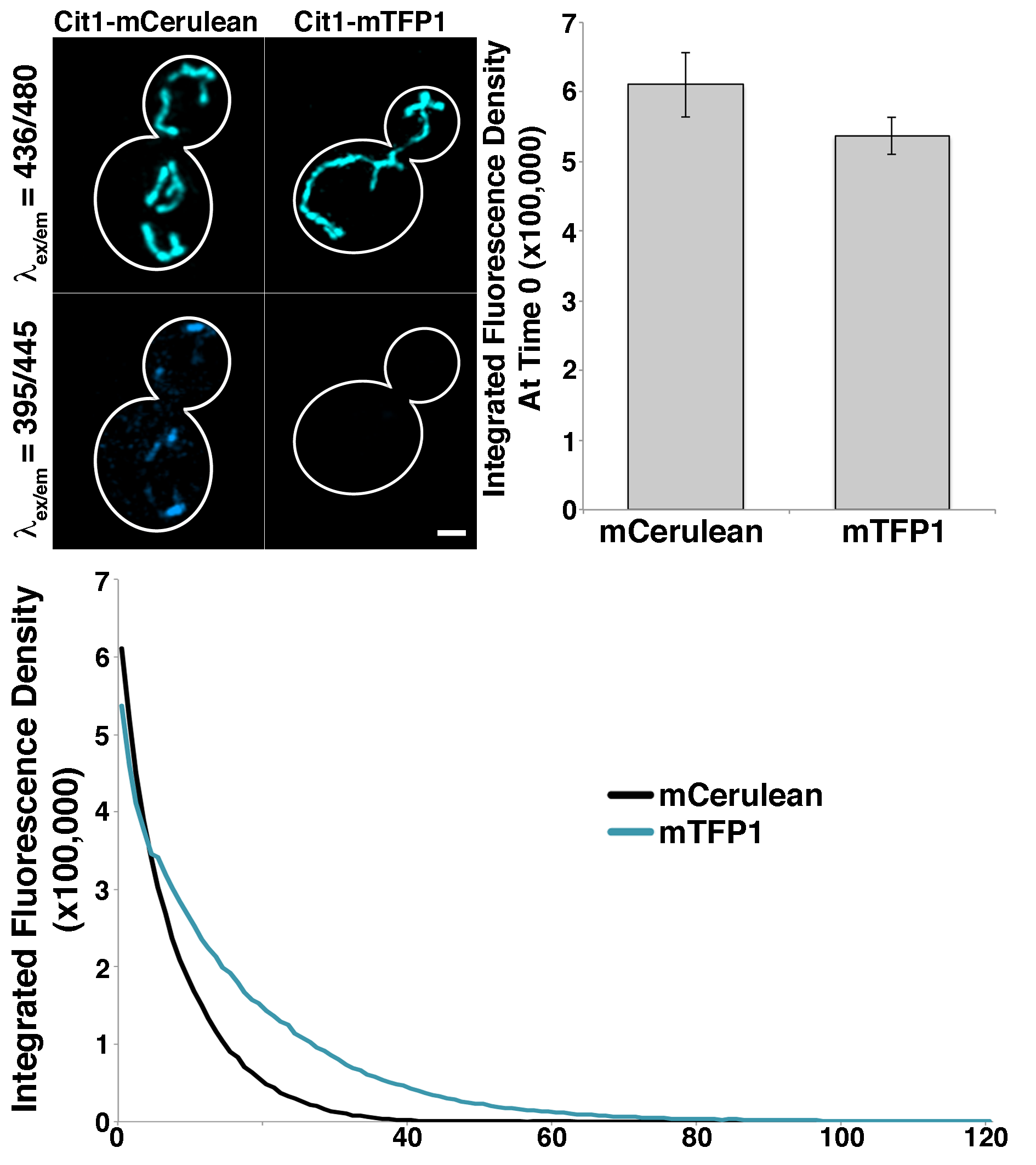

Supplement: S8 Fig — (A) BY4741 cells expressing Cit1-mCerulean or Cit1-mTFP1 were imaged on a wide-field microscope. Z-series were collected at 0.5 μm intervals throughout the entire cell using a metal-halide lamp with standard CFP and DAPI filters using 216 gain and 200 ms exposure times. Full filter specifications are listed in Materials and Methods. Only the center wavelengths of our filter settings for values of excitation and emission filters are listed for ease of readability. Scale bar is 1 μm. (B-C) BY4741 cells expressing Cit1-mTFP1 or Cit1-mCerulean were imaged on a wide-field microscope. Single-plane, time-lapse imaging was performed at the center of the cell at 1 sec intervals for 120 sec using a metal halide lamp and CFP filters at 216 gain and 200 ms exposure for each fluorophore. B) Integrated fluorescence density at time = 0 was measured using Image J as described in Materials and Methods. *** = p < 0.001. Error bars are SEM. C) Integrated fluorescence density was measured at each time point and normalized to the integrated fluorescence density at time = 0 and graphed as % fluorescence remaining as a function of time. Error bars are SEM. n = 35–39 cells per strain. Data is representative of 3 independent trials. (TIF) [file pone.0146120.s008.tif]
