# Supplementary material for: Characterization of Fluorescent Proteins for Three- and Four-Color Live-Cell Imaging in S. cerevisiae
Source: PLoS One. 2016 Jan 4;11(1):e0146120. doi: 10.1371/journal.pone.0146120 (PMC4699809; doi:10.1371/journal.pone.0146120)
Supplement: S1 File — Table A. Primers used to synthesize tagging vectors. Table B. Tagging vectors used in this study. Vectors synthesized in this study are available on Addgene. Table C. Primers used in this study to synthesize fluorescent protein fusions. Table D. Strains used in this study. (DOCX) [file pone.0146120.s009.docx]

**Supporting Information**

**S1 File**

| **Purpose** | **Forward** | **Reverse** |
| --- | --- | --- |
| mTFP1 into pFA6 | CCGGTTAATTAACAGTATGGTGAGCAAGGGCGAGGA | AATTGGCGCGCCTTACTTGTACAGCTCGTCCA |
| mTFP1 into pOM | GCAGGGATCCGTGAGCAAGGGCGAGGAGAC | GCAGACTAGTCTTGTACAGCTCGTCCATGCC |
| mCitrine into pOM | GCAGGGATCCTCTAAAGGTGAAGAATTATTACTGGTGTTGT | GCAGACTAGTTTTGTACAATTCATCAATACCATGGGTAATACC |
| mCherry into pOM | GCAGGGATCCGTGAGCAAGGGCGAGGAGGATAA | GCAGACTAGTCTTGTACAGCTCGTCCATGCC |

**Table A. Primers used to synthesize tagging vectors**

| **Plasmid** | **Purpose** | **Reference** |
| --- | --- | --- |
| pFA6 -GFP(S65T)-KanMX6 | C-terminal GFP tagging using *KanMX6* as a selection marker | [12] |
| pFA6 -GFP(S65T)-His3MX6 | C-terminal GFP tagging using *HIS3* as a selection marker | [12] |
| pFA6 -mTFP1-KanMX6 | C-terminal mTFP1 tagging using *KanMX6* as a selection marker | This study |
| pFA6 -mTFP1-His3MX6 | C-terminal mTFP1 tagging using *HIS3* as a selection marker | This study |
| PCY3090-02 | C-terminal mCherry tagging using *hphMX4* as a selection marker | [13] |
| PCY3080-07 | C-terminal mCitrine tagging using *Sh ble* as a selection marker | [13] |
| pOM42 | N-terminal GFP tagging using *LEU2* as a selection marker | [11] |
| pOM43 | N-terminal GFP tagging using *URA3* as a selection marker | [11] |
| pOM42-mTFP1 | N-terminal mTFP1 tagging using *LEU2* as a selection marker | This study |
| pOM43-mTFP1 | N-terminal mTFP1 tagging using *URA3* as a selection marker | This study |
| pOM42-mCitrine | N-terminal mCitrine tagging using *LEU2* as a selection marker | This study |
| pOM43-mCitrine | N-terminal mCitrine tagging using *URA3* as a selection marker | This study |
| pOM42-mCherry | N-terminal mCherry tagging using *LEU2* as a selection marker | This study |
| pOM43-mCherry | N-terminal mCherry tagging using *URA3* as a selection marker | This study |

**Table B. Tagging vectors used in this study.**

| **Gene** | **Vector** | **Forward** | **Reverse** |
| --- | --- | --- | --- |
| *CIT1* | pFA6 | AAAATACAAGGAGTTGGTAAAGAAAATCGAAAGTAAGAACCGGATCCCCGGGTTAATTAA | AATAGTCGCATACCCTGAATCAAAAATCAAATTTTCCTTAGAATTCGAGCTCGTTTAAAC |
| *CIT1* | PCY | AAAATACAAGGAGTTGGTAAAGAAAATCGAAAGTAAGAACGGTGACGGTGCTGGTTTA | TTTGAATAGTCGCATACCCTGAATCAAAAATCAAATTTTCCATCGATGAATTCGAGCTCG |
| *CIT1* | pOM | AAAATATGCAAAAGGCTCTTTTTGCACTATTGAATGCTCGCCACTATAGTAGCTGCAGGTCGACAACCCTTAAT | TCTGCCTTTGCTGGGATAATTTCAGCAAATCTCTCCTTCAACGTTTGTTCGGAGGCGCGGCCGCATAGGCGACT |
| *PHO88* | PCY | AGAAGCTGAAAGAGCCGGTAACGCTGGTGTTAAGGCTGAAGGTGACGGTGCTGGTTTA | AAAACTAGGAAAAAAAAATACTTCGCTTTTGATCGAATCAATCGATGAATTCGAGCTCG |
| *ERG6* | PCY | GAAAACGCCGAAACCCCCTCCCAAACTTCCCAAGAAGCAACTCAAGGTGACGGTGCTGGTTTA | ATCTGCATATATAGGAAAATAGGTATATATCGTGCGCTTTATTTGATCGATGAATTCGAGCTCG |

**Table C. Primers used in this study to synthesize fluorescent protein fusions.**

| **Strains** | **Genotype** | **Source** |
| --- | --- | --- |
| BY4741 | *MATa his3∆1 leu2∆0 met15∆0 ura3∆0* | Open Biosystems |
| RHY116 | *MATa his3∆1 leu2∆0 met15∆0 ura3∆0 CIT1-GFP(S65T)-HIS3* | This study |
| RHY220 | *MATa his3∆1 leu2∆0 met15∆0 ura3∆0 CIT1-yEpolylinker-mCherry-hphMX4* | This study |
| RHY225 | *MATa his3∆1 leu2∆0 met15∆0 ura3∆0 CIT1-yEpolylinker-yEmCitrine-Sh ble* | This study |
| RHY301 | *MATa his3∆1 leu2∆0 met15∆0 ura3∆0 CIT1-mTFP1-HIS3* | This study |
| RHY303 | *MATa his3∆1 leu2∆0 met15∆0 ura3∆0 CIT1-mTFP1-HIS3 Pho88-yEpolylinker-yEmCitrine-Sh ble Erg6-yEpolylinker-mCherry-hphMX4* | This study |
| RHY349 | *MATa his3∆1 leu2∆0 met15∆0 ura3∆0 yEmCitrine-CIT1* | This study |
| RHY360 | *MATa his3∆1 leu2∆0 met15∆0 ura3∆0 mCherry-CIT1* | This study |
| RHY366 | *MATa his3∆1 leu2∆0 met15∆0 ura3∆0 mTFP1-CIT1* | This study |
| RHY453 | *MATa his3∆1 leu2∆0 met15∆0 ura3∆0 CIT1-mCerulean-Sh ble* | This study |

**Table D. Strains used in this study.**
